# Supplementary material for: Ensemble Docking as a Tool for the Rational Design of Peptidomimetic Staphylococcus aureus Sortase A Inhibitors
Source: Int J Mol Sci. 2024 Oct 20;25(20):11279. doi: 10.3390/ijms252011279 (PMC11508331; doi:10.3390/ijms252011279)
Supplement: Supplementary file 1 [file ijms-25-11279-s001.zip › supporting_info/supplementary.pdf]

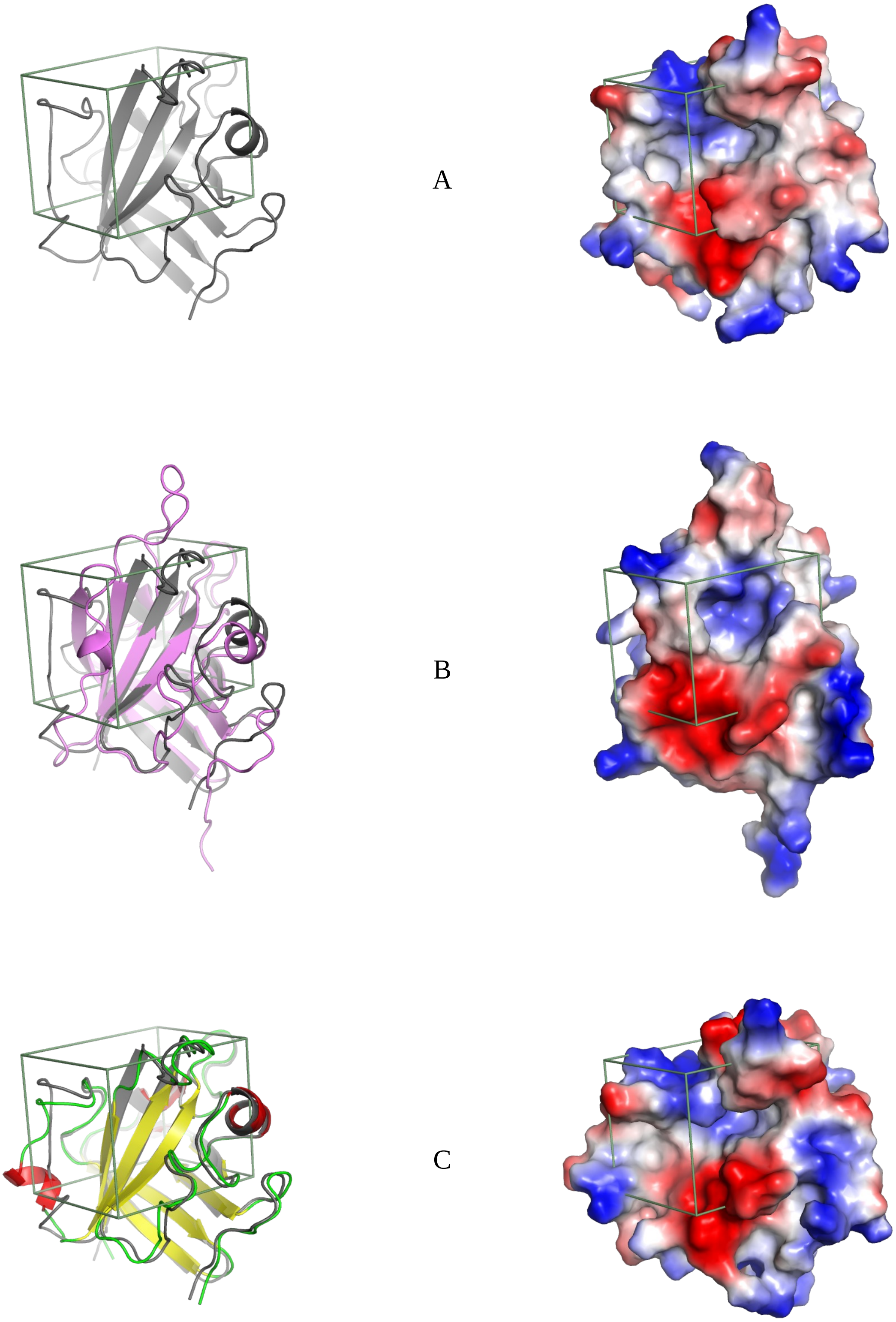
A
B
C

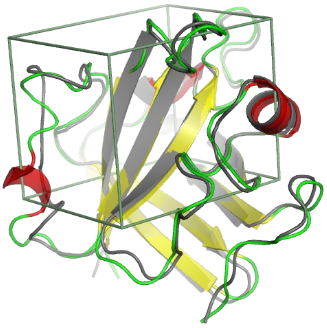

D

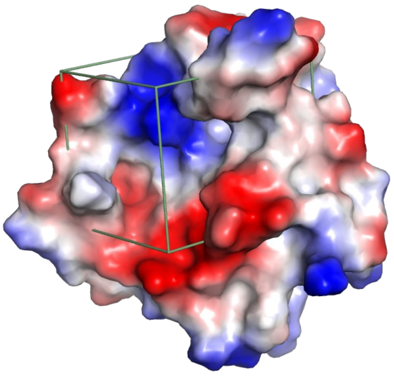

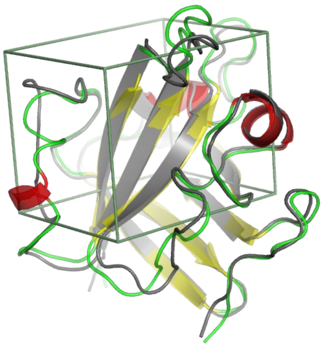

E

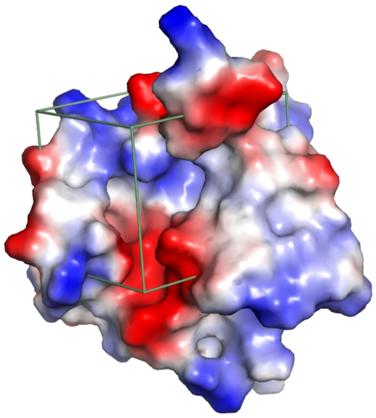

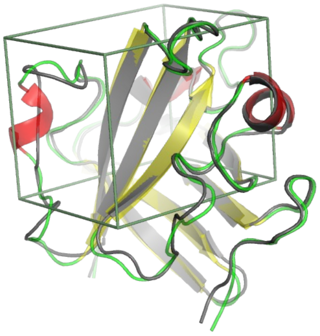

F

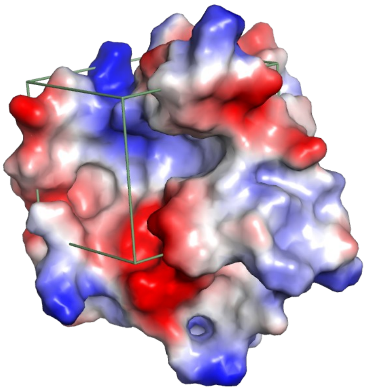

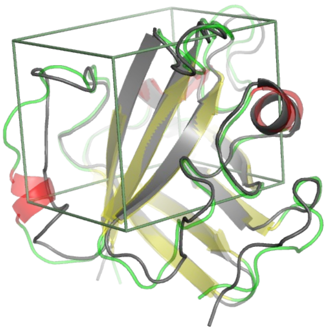

G

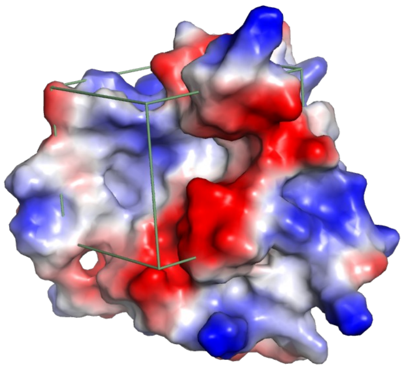

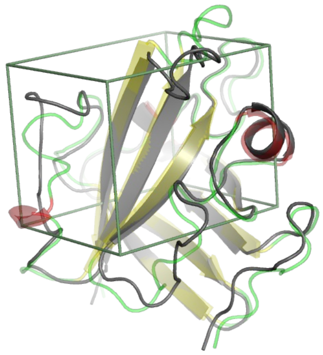

H

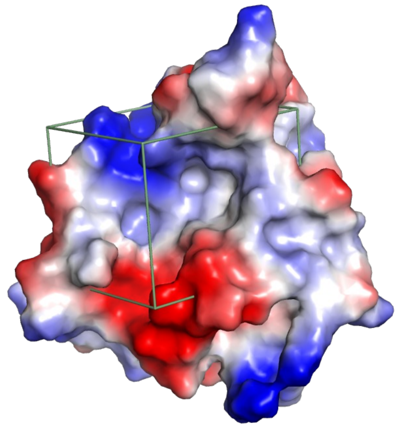

Figure S1. Depiction of the grid bounding box used for docking for each of the protein structures used: A – PDB:1T2W (the main template), B – PDB:2KID, C – Cluster #1, D – Cluster #2, E – Cluster #3, F – Cluster #4, G – Cluster #5, H – Cluster #6. Ribbon models (left column) are superimposed over PDB:1T2W geometry for reference which is shown in grey.
